# Optimisation of Phenolic Compound Extraction from *Agrimonia eupatoria* L. Using Response Surface Methodology for Enhanced Yield of Different Phenolics and Maximised Antioxidant Activity

**DOI:** 10.3390/antiox14070831

**Published:** 2025-07-07

**Authors:** Justinas Sukackas, Modestas Žilius, Gerda Šaltytė, Lina Raudonė

**Affiliations:** 1 Department of Pharmacognosy, Lithuanian University of Health Sciences, Sukileliu av. 13, LT-50162 Kaunas, Lithuania; justinas98@gmail.com (J.S.); gerda.saltyte@stud.lsmu.lt (G.Š.); 2 Institute of Pharmaceutical Technologies, Lithuanian University of Health Sciences, Sukileliu av. 13, LT-50162 Kaunas, Lithuania; modestas.zilius@lsmu.lt; 3 Department of Clinical Pharmacy, Lithuanian University of Health Sciences, Sukileliu av. 13, LT-50162 Kaunas, Lithuania

**Keywords:** *Agrimonia eupatoria* L., agrimoniin, antioxidant, extraction, response surface methodology, phenolics

## Abstract

*Agrimonia eupatoria* L. is a traditionally used medicinal plant rich in tannin compounds with antioxidant, anti-inflammatory, and antimicrobial activities. This study aimed to optimise the extraction of individual phenolic acids, flavonoids, and tannins from *A. eupatoria* and maximise their antioxidant activity using response surface methodology (RSM). A central composite design was applied to evaluate the influence of acetone concentration, solvent ratio, and extraction time on the yield of total phenolics, total radical scavenging and reducing capacities, and individual compounds. Acetone concentration, solvent ratio, and extraction time were varied in a central composite design. The optimal conditions yielded high levels of agrimoniin (9.16 mg/g), total identified phenolics (33.61 mg/g), and strong antioxidant activity. These findings provide a scientific basis for standardising bioactive-rich extracts for nutraceutical and pharmaceutical applications.

## 1. Introduction

*Agrimonia eupatoria* L. (agrimony) is a perennial herbaceous plant of the Rosaceae Juss. family, native to the Northern Hemisphere and widely distributed in Europe, Asia, Africa, and North America. The plants naturally grow in meadows and pastures, flowering from June to September [1]. *Agrimonia eupatoria* has been traditionally used in folk medicine for its anti-inflammatory effects and in the treatment of diarrhoea, inflammatory conditions of the mouth and throat, mucous membrane irritations, skin ailments, wounds, and disorders affecting the liver and lungs [1]. The aboveground parts are included in British, Czech, and European Pharmacopoeias, and in the latter, their quality has been defined as the total amount of tannin content [2]. On the other hand, besides tannins, raw materials from agrimony contain various biologically active compounds, including flavonoids, phenolic acids, triterpenoids, essential oils, minerals, monosaccharides, and vitamins. These compounds are known for their diverse pharmacological activities, such as antioxidant, anti-inflammatory, antiviral, antibacterial, analgesic, and hepatoprotective effects [3].

Studies have revealed the diverse pharmacological effects of *Agrimonia eupatoria*. *Agrimonia* is known to have anti-inflammatory, antibacterial, antiviral and antioxidative effects, while some research suggests it may help control blood sugar levels and have anticancer and hepatoprotective potential [1,3,4,5]. The antioxidative properties of *Agrimonia* are attributed to its phenolic compounds, such as phenolic acids, flavonoids, ellagitannins, and flavan-3-ol derivatives [3]. The polyphenol-rich fractions of *Agrimonia*, especially those high in quercetin, kaempferol, apigenin, and luteolin derivatives, have been shown to have antioxidant and anti-inflammatory effects [6,7,8]. The antidiabetic effect is considered to be related to the abundance of procyanidins and flavonol glycosides in *A. eupatoria*, since plant matter extracts demonstrate good anti-glucosidase, anti-glycation, and anti-hyperglycaemic effects, as well as good aldose reductase inhibitory activity [9,10]. *Agrimonia* extracts have been the subject of studies considering neuropathic pain. Research findings suggest that *A. eupatoria* extract-induced rats showed a higher antinociceptive property than those induced with gabapentin [11].

Different extracts of *Agrimonia* have been shown to have varying effects on bacterial resistance to ampicillin, with the water extract being the most effective and the acetone extract being the least [12]. The antibacterial effects of the plant extract were also found to have an impact on wound treatment and the promotion of their healing [13]. In addition, the comparison of different types of *Agrimonia* extracts also showed varying results of antioxidant and reducing power activities, which correlated with the phenolic content, flavonoid, tannin, and proanthocyanidin concentrations of the mentioned extracts [8,14]. Research has already demonstrated that various factors during extraction have distinct impacts on the quantitative properties of phenolic compounds and the antioxidant activity of *Agrimonia* extracts [15]. Since these bioactive compounds contribute to the diverse therapeutic effects of *Agrimonia*, making it a valuable plant matter in traditional and modern herbal medicine, it is crucial to optimise extraction methods of the mentioned compounds from the *Agrimonia eupatoria* plant matrix.

Phenolic compounds are particularly interesting due to their notable antioxidant activity, and they participate in the pathogenesis mechanisms of various diseases, including cardiovascular diseases, cancers, and neurodegenerative disorders [16]. Furthermore, it is important to determine the profile of phenolic compounds and their antioxidant activity to develop effective strategies for preventing and treating these diseases. Selecting the optimal conditions for the extraction process of these compounds can shift the preparation of standardised extracts towards the targeted individual phytochemical composition, enhance the therapeutic potential, and lead to the development of new pharmacological agents and functional ingredients [17,18].

Response surface methodology (RSM) is commonly applied to determine optimal extraction conditions [19]. This particular methodology employs mathematical and statistical techniques that consider multiple variables. It is particularly effective for optimising processes where multiple interacting factors must be considered. Using RSM, the relationships between various extraction parameters—such as solvent concentration, extraction time, material-to-solvent ratio, temperature, and others—can be evaluated by targeting the yield of phytochemical compounds of interest [20]. The application of RSM in this study ensures a systematic and efficient approach to optimising the most efficient antioxidant and potentially pharmacological effects of *Agrimonia eupatoria* extracts, leading to reliable and reproducible results, which are essential for scientific research and industrial applications [21,22].

This study aims to optimise the extraction conditions of *Agrimonia eupatoria* to target individual phenolic compounds and maximise their antioxidant activity. Identification of the optimal extraction parameters could contribute to the scientific understanding of *Agrimonia eupatoria* and its potential applications in pharmaceuticals, nutraceuticals, and the food industry. Despite previous studies on *Agrimonia eupatoria* [3,4,6,9,23], there is still a significant gap in comprehensive optimisation that addresses the extraction efficiency of individual compounds and the influence of different methods. This study aims to fill this gap by providing detailed phenolic profiling and assessing the antioxidant activity of the extracts under optimised conditions.

## 2. Materials and Methods

### 2.1. Chemicals and Solvents

The solvents, 99.9% acetonitrile, 99.9% methanol, 99.8% anhydrous acetic acid, and 37% hydrochloric acid, and acetone, were obtained from Sigma-Aldrich (Steinheim, Germany); 99.8% trifluoroacetic acid was from Merck (Darmstadt, Germany). The following standard materials and reagents were obtained from Sigma-Aldrich: chlorogenic acid, neochlorogenic acid, 4-O-caffeoylquinic acid, ellagic acid, catechin, epicatechin, epigallocatechin (EGC), epicatechin gallate (ECG), epigallocatechin gallate (EGCG), procyanidin B1, procyanidin B2, hyperoside, *p*-coumaric acid, luteolin-7-glucoside, luteolin-7-glucuronide, isovitexin, quercetin-3-O-malonyl-glucoside, isoquercitrin, astragalin, quercitrin, tiliroside, kaempferol, and apigenin. Agrimoniin was purchased from LeapChem (Hong Kong). Sodium carbonate, Folin–Ciocalteu reagent, 6-hydroxy-2,5,7,8-tetramethylchroman-2-carboxylic acid (Trolox), 2,2-azino-bis(3-ethylbenzothiazoline-6-sulfonic acid) diammonium salt (ABTS), 2,4,6-tri-(2-pyridyl)-S-triazine (TPTZ), ferric chloride (FeCl3), and sodium acetate were obtained from Sigma-Aldrich (Steinheim, Germany). Potassium persulfate was obtained from Alfa Aesar (Karlsruhe, Germany).

The purified water was prepared using the Milli–Q (Millipore, Bedford, MA, USA) water purification system.

### 2.2. Plant Material and Preparation of Extracts

**Figure 1:**
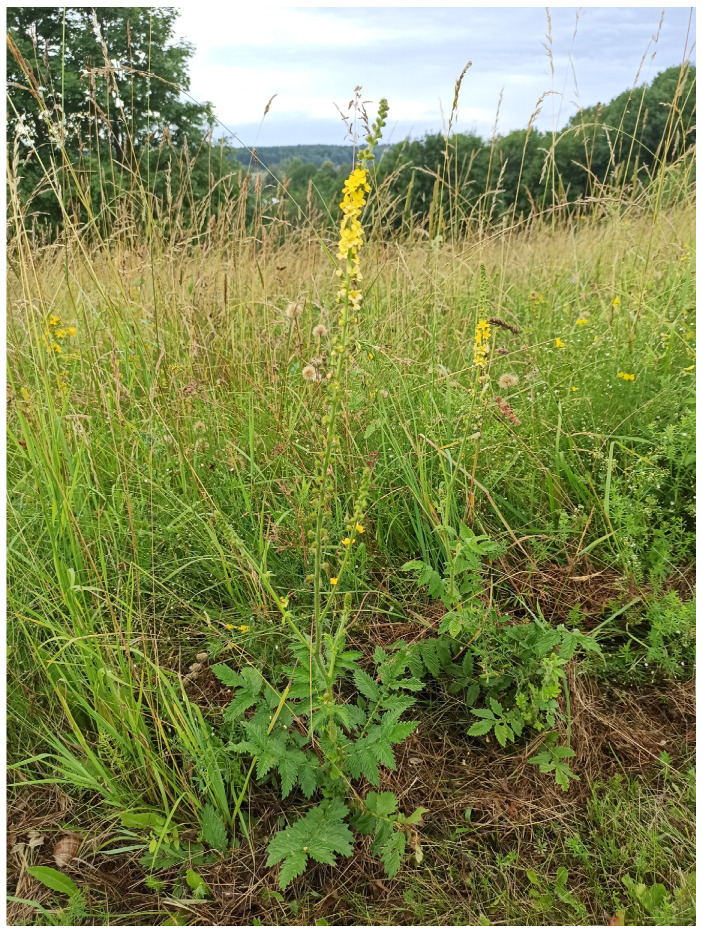
*Agrimonia eupatoria* in grassland. Photo by authors.

The aboveground parts of *A. eupatoria* were sampled from a natural population (Figure 1) in the Kaunas district, Lithuania (54.909630, 23.811343), during the massive flowering period in August 2022. Plant material was dried at room temperature and protected from direct sunlight. The air-dried plant material samples were grounded to a homogenous powder using an “IKA A11 basic miller” (“IKA-Werke Gmbh & Co. KG”, Staufen im Breisgau Germany). Milled powders were sieved using a “Retsch AS 200” (“Retsch Gmbh”, Haan, Germany) 355 µm sieve and placed in sealed dark glass containers. The loss of all plant material samples due to drying was determined and evaluated according to the method described in the European Pharmacopoeia (Ph. Eur. 2.2.32) [24], and the results were expressed on a dry weight (DW) basis. For each extraction, 1.0 g (precise weight) of milled and sieved plant material was placed into dark glass bottles. The corresponding solvent mixtures, prepared according to the central composite design (Table 1), were added in the specified solvent ratios (*w*/*v*). The bottles were tightly sealed. Samples were then placed in an ultrasonic bath and extracted for the designated periods (Table 1) at room temperature. Following extraction, the mixtures were centrifuged for 15 min at 6500 rpm. The resulting supernatants were filtered through cotton wool into wide-necked, dark glass bottles, sealed, labelled, and stored at 4 °C until further analysis.

### 2.3. Development of Extraction Process

#### Experimental Design for Modelling and Optimisation of Extraction Process

The central composite design (CCD) model was selected as the response surface experimental design (Design-Expert 13 (Stat-Ease, Inc., Minneapolis, MI, USA)). Acetone concentration, solvent ratio, and extraction time were selected as independent variables (factors) with the following limits: 0–100% acetone concentration, 10:1–100:1 solvent ratio, and 5–45 min extraction time. Within the limits of the specified conditions, 15 extraction conditions were employed (Table 1). The prepared extracts were passed through 0.22 µm pore size syringe filters (Carl Roth GmbH, Karlsruhe, Germany) and kept at 4 °C until analyses.

The efficiency of the extraction process was evaluated based on the parameters of the produced extracts, including total phenolic content, the amounts of phenolic acids, tannins, flavonoids, and agrimoniin, and radical scavenging and reducing activities.

### 2.4. Determination of Individual Phenolic Profile in A. eupatoria

HPLC-PDA (high-performance liquid chromatography) analysis was performed on a “Waters e2695 Alliance system” (Waters, Milford, MA, USA) equipped with a photodiode array detector “Waters 2998”. The “ACE Super C18” (ACT, UK) column (C18, 250 mm × 4.6 mm, particle size 3 μm) was maintained at 15 °C. The mobile phase consisted of A (0.05% trifluoracetic acid) and B (acetonitrile). The gradient scheme was as follows: 0 min, 85% A; 0–30 min, 70% A; 30–50 min, 40% A; 50–56 min, 10% A; 56–65 min, 15% A. The flow rate was 0.5 mL/min with an injection volume of 10 μL. Chromatographic peak identification was performed with reference to compounds and by comparing their retention times and UV absorption spectra at 200–400 nm with those of the analytes and reference compounds. Validation of the method was performed according to ICH Q2 (R2) guidelines. The parameters are presented in Table S1.

### 2.5. Determination of Total Phenolic Content and ABTS, and FRAP Activity Assays

We used the Folin–Ciocalteu method of Slinkard and Singleton 1977 modified by Kaunaite et al. in 2022 [25,26].

The radical scavenging activity was analysed using the ABTS radical cation decolourisation assay (ABTS), and the reducing activity was analysed using ferric reducing antioxidant power (FRAP). The ABTS radical cation assay is described by Re et al., whereas FRAP was performed using the method described by Benzie and Strain [27,28] with the modifications as in Raudone et al. (2019) [29] using a spectrophotometer (Spectronic CamSpec M550, Garforth, UK). The results were expressed as gram of dry weight of plant material in μmol TE/g.

### 2.6. Statistical Analysis

The data were processed using Microsoft Office Excel for 365 Version 2403 (Microsoft, Redmond, WA, USA) and SPSS 29 Version 29.0.1.0 (171) (IBM, Armonk, NY, USA) software. Experimental design data were evaluated by using the ANOVA statistical method when *p* < 0.05. The Design-Expert program, a surface response methodology design comprising 15 experiments, was created to help determine the optimal concentration of the extraction solvent, the proportion of raw material to solvent, and the time. The influence of the three variables on amounts of individual phenolic acids from the phytochemical profile was evaluated. Spearman’s correlation coefficients were calculated to assess the relationships between extraction parameters, individual phenolic compounds, total identified phenolic content, and antioxidant activity. The correlation strength was interpreted as weak (|r| < 0.3), moderate (0.3 ≤ |r| < 0.7), or strong (|r| ≥ 0.7), and a heatmap was constructed. All extractions and determinations were performed in triplicate. The data were expressed as the mean ± standard error.

## 3. Results

### 3.1. Assessment of Total Phenolic Content

**Figure 2:**
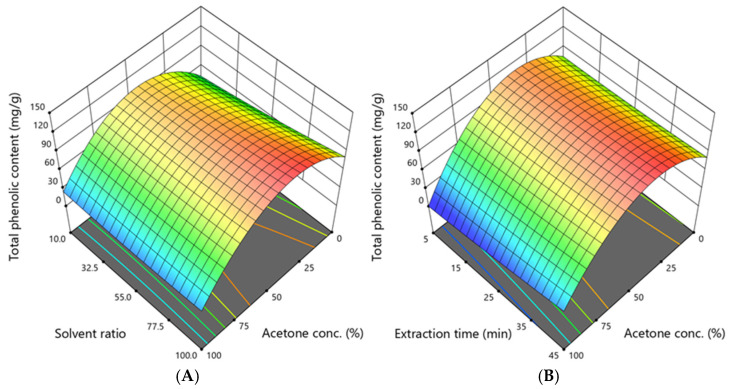
The dependence of the total phenolic content (TPC, mg GAE/g DW) extracted from *A. eupatoria* on (**A**)—the acetone concentration and solvent ratio when the extraction time was 45 min; (**B**)—the acetone concentration and extraction time when the solvent ratio was 100:1. The confidence interval (95%) for the mean is 73.19–133.65 mg/g.

The total phenolic content ranged from 10.65 to 120.60 mg/g, and its dependence on the acetone concentration, solvent ratio, and extraction time is illustrated in Figure 2.

The graphs illustrate that the total phenolic content is primarily dependent on the concentration of acetone. The highest amount of these compounds was obtained when the acetone concentration was 50%, the solvent ratio was 100:1, and the extraction time was 25 min. However, to extract a high total phenolic content, as predicted by the model, the acetone concentration should be 31–49%, the solvent ratio should be 95–100:1, and the extraction time should be 41–45 min.

### 3.2. Assessment of the Amount of Phenolic Acids

**Figure 3:**
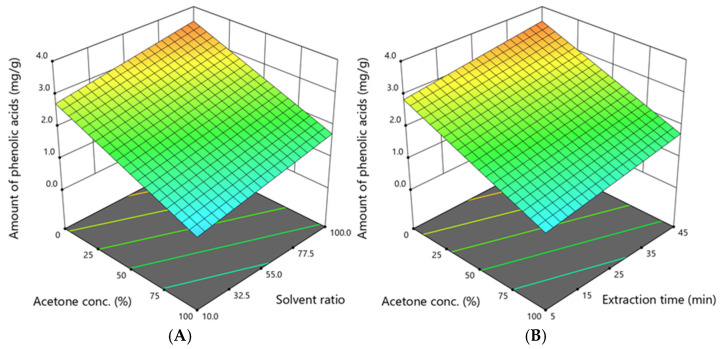
The dependence of the amount of phenolic acids (mg/g DW) extracted from *A. eupatoria* on (**A**)—the acetone concentration and solvent ratio when the extraction time was 45 min; (**B**)—the acetone concentration and extraction time when the solvent ratio was 100:1. The confidence interval (95%) for the mean is 1.4–2.4 mg/g.

The total amount of determined phenolic acids, including neochlorogenic acid, chlorogenic acid, ellagic acid, p-coumaric acid, and 4-O-caffeoylquinic acid, ranged from 0.210 to 3.852 mg/g, and their dependence on acetone concentration, solvent ratio, and extraction time is shown in Figure 3.

The graphs illustrate that the amount of phenolic acids depends on the acetone concentration, solvent ratio, and extraction time. The highest amount of these acids was obtained when the acetone concentration was 0%, the solvent ratio was 55:1, and the extraction time was 25 min. However, to extract a high amount of phenolic acids, as predicted by the model, the acetone concentration should be 0–5%, the solvent ratio should be 90–100:1, and the extraction time should be 41–45 min.

### 3.3. Assessment of the Amount of Flavan-3-ols and Tannins

**Figure 4:**
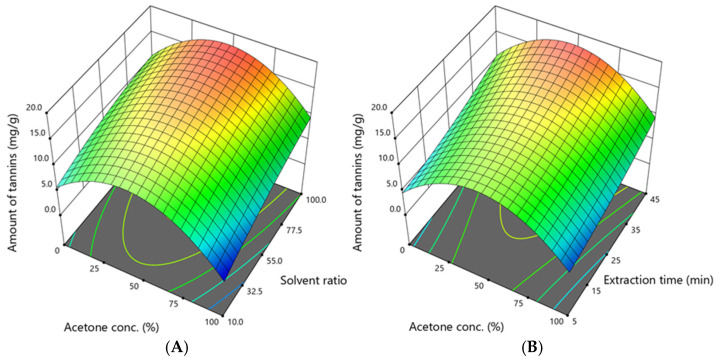
The dependence of the total amounts of agrimoniin, catechin, epicatechin, and procyanidins B1 and B2 (as representatives of flavan-3-ols and condensed tannins) (mg /g DW) extracted from *A. eupatoria* on (**A**)—the acetone concentration and solvent ratio when the extraction time was 45 min; (**B**)—the acetone concentration and extraction time when the solvent ratio was 100:1. The confidence interval (95%) for the mean is 7.8–17.9 mg/g.

In this study, flavan-3-ols (catechin, epicatechin, procyanidins B1 and B2) were grouped with tannins, as they form the structural units of condensed tannins (proanthocyanidins). Agrimoniin, the predominant compound in *A. eupatoria*, is classified as a hydrolysable ellagitannin [30]. This classification reflects the broader tannin group, which includes both hydrolysable and condensed forms, and was applied to evaluate their extraction behaviour. The total amount of determined tannins and flavan-3-ols, including agrimoniin, catechin, epicatechin, procyanidin B1 procyanidin B2, GCG, and EGCG, was in a range of 1.580 to 17.863 mg/g, and the dependence on acetone concentration, solvent ratio, and extraction time is shown in Figure 4.

Yields peaked under similar conditions to those of total phenolics—50% acetone, a 100:1 solvent ratio, and 25 min. As shown in Figure 3, optimal extraction was achieved with a solvent ratio of 91–100% and an extraction time of up to 45 min, confirming the need for moderate polarity and a high solvent volume.

### 3.4. Assessment of the Sum of Identified Flavones and Flavonols

**Figure 5:**
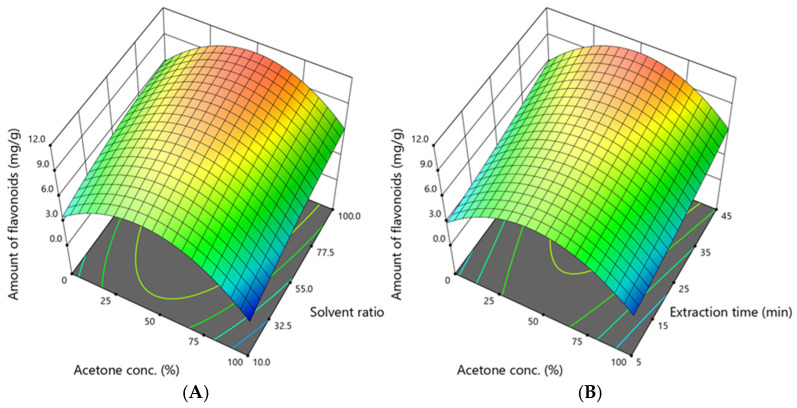
The dependence of the sum of determined flavonoids from flavonol and flavone subclasses (mg /g DW) extracted from *A. eupatoria* on (**A**)—the acetone concentration and solvent ratio when the extraction time was 45 min; (**B**)—the acetone concentration and extraction time when the solvent ratio was 100:1. The confidence interval (95%) for the mean is 5.0–11.5 mg/g.

The identified amounts of flavonols and flavones (quercitrin, isoquercitrin, kaempferol, hyperoside, tiliroside, astragalin, quercetin-3-*O*-malonyl-glc, apigenin, isovitexin, luteolin-7-glucuronide, luteolin-7-glucoside) were summed up to calculate the flavonoid amount. The amount of flavonoids was 0.924–11.178 mg/g, and the dependence on acetone concentration, solvent ratio, and extraction time is shown in Figure 5.

Response surface analysis revealed that optimal recovery occurred within a narrow range of 45–55% acetone, a solvent ratio of 95–100:1, and an extraction time of approximately 45 min. These conditions align closely with those favouring tannin extraction, suggesting similar solvent affinity for these two phenolic groups. As shown in Figure 4, acetone concentration contributed more substantially than extraction time or solvent volume, consistent with the moderately lipophilic nature of flavonoid aglycones and glycosides. Overall, the trends in flavonoid yield corresponded to those of total phenolics and tannins, reinforcing the role of intermediate solvent polarity and high solvent availability in the efficient extraction of polyphenols.

### 3.5. Assessment of the Amount of Agrimoniin

**Figure 6:**
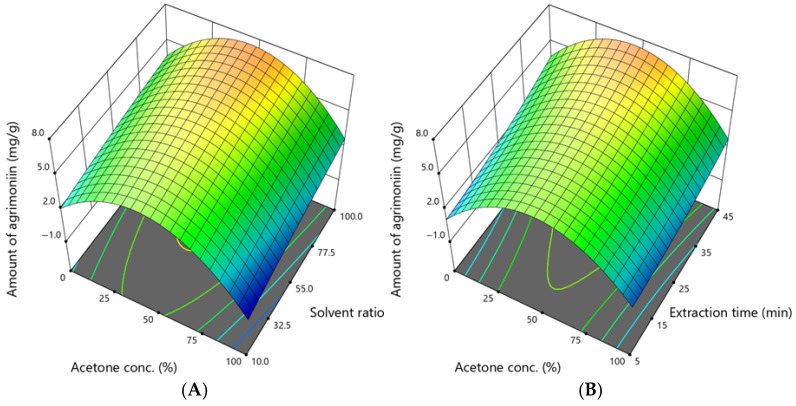
The dependence of the amount of agrimoniin (mg /g DW) extracted from *A. eupatoria* on (**A**)—the acetone concentration and solvent ratio when the extraction time was 45 min; (**B**)—the acetone concentration and extraction time when the solvent ratio was 100:1. The confidence interval (95%) for the mean is 2.7–7.7 mg/g.

Agrimoniin was evaluated individually as the key predominant compound in the profile. The amounts of agrimoniin ranged from 0.056 to 8.265 mg/g, and the dependence on acetone concentration, solvent ratio, and extraction time is shown in Figure 6.

The graphs illustrate that the amount of agrimoniin depends mainly on the acetone concentration. The highest amount of this compound was obtained when the acetone concentration was 50%, the solvent ratio was 100:1, and the extraction time was 25 min. However, to extract a high amount of agrimoniin, as predicted by the model, the acetone concentration should be 36–57%, the solvent ratio should be 95–100:1, and the extraction time should be 45 min.

### 3.6. Assessment of Antioxidant Activity

**Figure 7:**
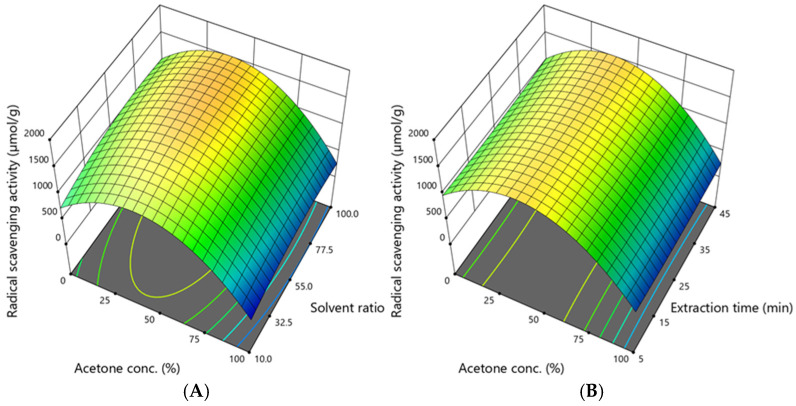
The dependence of radical scavenging activity (µmol/g DW) on (**A**)—the acetone concentration and solvent ratio when the extraction time was 45 min; (**B**)—the acetone concentration and extraction time when the solvent ratio was 100:1. The confidence interval (95%) for the mean is 970–1924 µmol/g.

The radical scavenging activity ranged from 93.12 to 1865.06 µmol/g, and its dependence on acetone concentration, solvent ratio, and extraction time is shown in Figure 7.

The graphs illustrate that the radical scavenging activity depends mainly on the acetone concentration. The highest activity was obtained when the acetone concentration was 50%, the solvent ratio was 100:1, and the extraction time was 25 min. However, to achieve a high radical scavenging activity, as predicted by the model, the acetone concentration should be 34–44%, the solvent ratio should be 67–86:1, and the extraction time should be 45 min.

**Figure 8:**
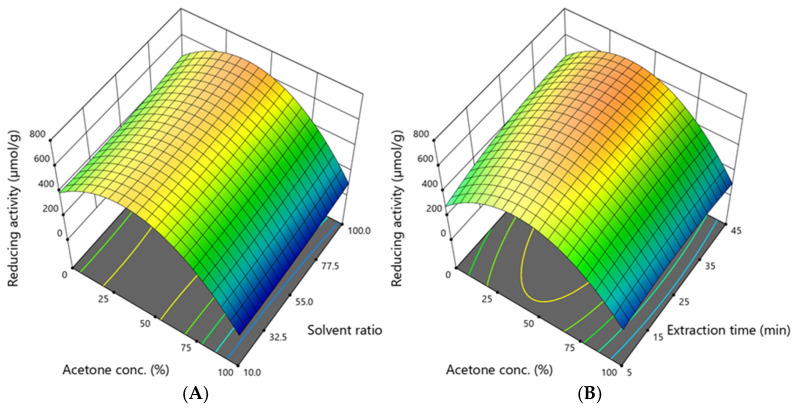
The dependence of reducing activity (µmol/g DW) on (**A**)—the acetone concentration and solvent ratio when the extraction time was 45 min; (**B**)—the acetone concentration and extraction time when the solvent ratio was 100:1. The confidence interval (95%) for the mean is 472–687 µmol/g.

The reducing activity ranged from 39.42 to 735.50 µmol/g, and its dependence on acetone concentration, solvent ratio, and extraction time is shown in Figure 8.

The graphs illustrate that the reducing activity depends mainly on the acetone concentration. The highest activity was obtained when the acetone concentration was 50%, the solvent ratio was 100:1, and the extraction time was 25 min. However, to achieve the high reducing activity that the model predicted, the acetone concentration should be 34–44%, the solvent ratio should be 100:1, and the extraction time should be 32–40 min. Both activities reached their highest levels at a 50% acetone concentration, a 100:1 solvent ratio, and a 25-min duration. The optimal ranges (34–44% acetone, 67–100:1 solvent ratio, 32–45 min) correspond to the conditions that favour the extraction of flavonoids and tannins (Figure 6 and Figure 7). These results reinforce the close link between phenolic content and antioxidant capacity.

Overall, it can be stated that to extract high amounts of phenolic compounds of distinct chemical origin and maximise their antioxidant activity, as predicted by the model, the acetone concentration should be 34–46%, the solvent ratio should be 95–100:1, and the extraction time should be 32–45 min. Then, the total phenolic content would be 119.52–121.95 mg/g, the amount of phenolic acids would be 2.69–2.93 mg/g, the amount of tannins and flavan-3-ols would be 17.16–17.76 mg/g, the amount of flavonols and flavones would be 10.37–10.83 mg/g, the amount of agrimoniin would be 7.11–7.37 mg/g, the radical scavenging activity would be 1522.09–1553.21 µmol/g, and the reducing activity would be 636.31–653.72 µmol/g. Comparing these predicted values of the studied parameters with the experimentally obtained maximum values, it can be seen that the total phenolic content will suffer a 0.9% loss or even a 1.1% gain. Next, the losses will be 0.6–3.9% for tannins, 3.1–7.2% for flavonoids (including flavonols and flavones), and 10.8–14.0% for agrimoniin. The most considerable losses (23.9–30.2%) will be for phenolic acids. When assessing antioxidant and reducing activities, the losses will reach 16.7–18.4% and 11.1–13.5%, respectively.

In practical applications, the availability of solvents and environmental concerns may limit the feasible solvent ratio [31]. To assess this impact, we used the model to predict yields at reduced ratios of 50:1 and 70:1. Compared to the optimal range (95–100:1), the total phenolic content was reduced by approximately 18% and 9%, respectively. For radical scavenging activity, reductions of 20–25% and 10–12% were estimated. These findings provide a reference point for selecting, if necessary, suboptimal but still reasonable conditions.

### 3.7. Statistical Analysis of Response Models via ANOVA

The fit of the model was assessed based on the *p*-value (*p* < 0.05). Statistical analysis data are presented in Table 2.

Based on the results obtained for each response (dependent variable), the linear model of the cumulative amount of phenolic acids and the quadratic model of reducing activity were statistically significant (*p* = 0.0382 and *p* = 0.0060, respectively).

The model terms are considered statistically significant if the *p*-value is lower than 0.05. When the *p*-value is greater than 0.10, such model terms are insignificant and can be removed from the equation, improving the model. These data are given in Table 3.

ANOVA showed that factor A (in some cases) and A^2^ are statistically significant in both significant and insignificant models. This suggests that the extraction of phenolic compounds is influenced solely by the acetone concentration. Meanwhile, the solvent ratio and extraction time do not affect this process. The models were simplified to remove insignificant terms. In this case, the model for each response was improved and became statistically significant. The data are presented in Table 4.

ANOVA showed that in some cases (cumulative amount of tannins, cumulative amount of flavonoids, amount of agrimoniin), factor A (acetone concentration) remains statistically insignificant (*p* = 0.1850, *p* = 0.2128, and *p* = 0.2232, respectively) even after simplifying the mathematical equation. However, factor A2, indicating the nonlinearity of the effect, is statistically significant in all cases.

The difference between the adjusted R-squared values confirms the fit of these models, with predicted R-squared values of less than 0.2 and an adequate precision value of greater than 4. The model of each response has a mathematical equation that incorporates actual factors used to make predictions. Statistical analysis data and the equations are presented in Table 5.

ANOVA showed that in all cases, Δ R^2^ is less than 0.2, and adequate precision is greater than 4. This indicates that all models are reliable, stable, and suitable for predictions. The higher the adjusted and predicted R^2^ values, i.e., approaching 1, the stronger, more reliable, and better balanced the model is, so it can be used for optimisation, predictions, or practical applications. As can be seen, the reducing activity quadratic model has a very good model fit and predictive power. The total phenolic content and radical scavenging activity quadratic models have good model fit and predictive power. The amount of tannins, the amount of flavonoids, and the amount of agrimonin follow quadratic models which have a moderately good model fit and predictive power. Meanwhile, the linear model for total phenolic acid amount has a weak model fit, and in this case, such model is not suitable for accurate predictions.

### 3.8. Phytochemical Profile and Antioxidant Activity of A. eupatoria Extracts Under Optimised Extraction Conditions

**Figure 9:**
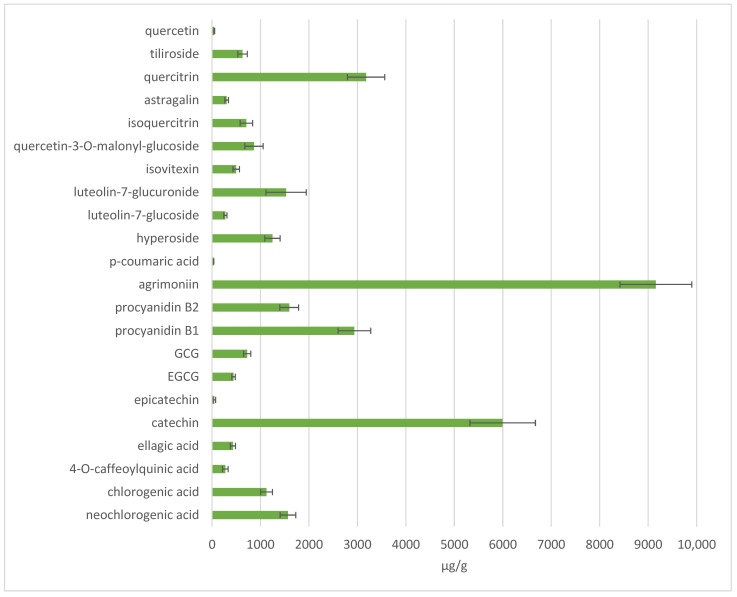
Quantitative profile (µg/g, DW) of phenolic compounds in aboveground parts of *A. eupatoria*.

To determine and evaluate the model predictions, an extract was prepared using 35% acetone, a 100:1 solvent ratio, and an extraction time of 30 min, conditions selected from the predicted range. The conditions were chosen to represent a balance between maximising extraction efficiency and potent antioxidant activity, and ensuring shorter times for routine application. Additionally, the use of 35% acetone favours both high-molecular-weight tannins and phenolic acids, making it suitable for multicomponent targeting. A quantitative analysis of phenolic compounds in the aboveground parts of *A. eupatoria* revealed a diverse and abundant profile of bioactive constituents (Figure 9). Agrimoniin was the predominant compound, reaching 9159.46 ± 746.32 µg/g of dry weight and accounting for 27% of the total quantified phenolics. This ellagitannin significantly exceeded the levels of other compounds, being the key compound and a contributor to the plant’s antioxidant potential. Among other identified compounds, catechin (5997.37 ± 675.50 µg/g; 18%) was the second prevailing compound, followed by quercitrin (3179.41 ± 386.23 µg/g; 9%) and procyanidin B1 (2937.31 ± 336.78 µg/g; 9%). Other identified compounds constituted 47% of all quantified compounds. These results confirm that *A. eupatoria* is particularly rich in ellagitannins and flavan-3-ols.

The antioxidant activity of *A. eupatoria* extract, assessed using the ABTS and FRAP assays, demonstrated strong radical scavenging and reducing capacities, with values of 1555.17 ± 345 µmol TE/g and 618.90 ± 154 µmol TE/g, respectively.

### 3.9. Correlation Analysis of Phenolic Compounds and Antioxidant Activity

**Figure 10:**
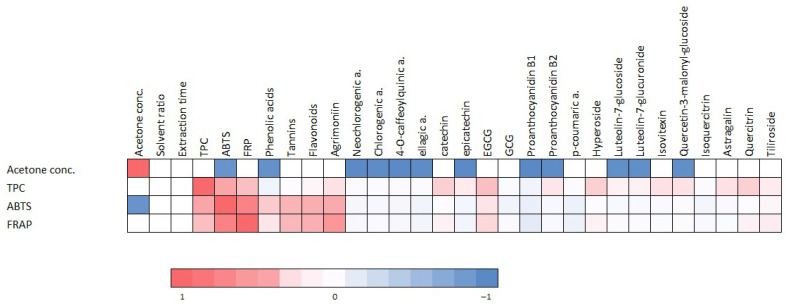
A heatmap of the correlation interdependencies between extraction condition parameters, individual phenolic compounds, and antioxidant activities (TPC—total phenolic compounds).

The correlation analysis revealed significant interrelationships between extraction parameters, individual phenolic compounds, and antioxidant activity (Figure 10). Among the extraction variables, acetone concentration showed a moderate negative correlation with total phenolic content (TPC, r = –0.529), radical scavenging activity (r = –0.529), and reducing activity (r = –0.567). This suggests that higher concentrations of acetone may reduce the efficiency of phenolic compound extraction and, consequently, the antioxidant potential. Total identified phenolic compounds (TPC) were strongly positively correlated with both radical scavenging activity (r = 0.893) and reducing activity (r = 0.850), confirming that phenolic compounds significantly contribute to the antioxidant potential of the extracts. Furthermore, radical scavenging and reducing activity were very highly correlated with each other (r = 0.957), indicating consistency across antioxidant assays. Several individual phenolic compounds exhibited strong positive correlations with antioxidant activities. Among them, agrimoniin demonstrated the strongest correlation with both radical scavenging (r = 0.886) and reducing activity (r = 0.921), suggesting that it may be a key contributor to the antioxidant properties of the extract. Other compounds with strong correlation interdependence included quercitrin, hyperoside, EGCG, catechin, astragalin, isovitexin, and quercetin-3-malonyl-glucoside (r values > 0.70 for all). On the other hand, several phenolic acids, including neochlorogenic, chlorogenic, and ellagic acid, were negatively correlated with acetone concentration (all r ≤ –0.624), indicating that these compounds are more efficiently extracted with a lower acetone content.

## 4. Discussion

This study presents a systematic optimisation of phenolic compound extraction from *Agrimonia eupatoria*, targeting key antioxidant constituents using response surface methodology (RSM). This approach offers a novel framework for maximising compound yield and potential bioactivity, advancing previous work that primarily focused on total phenolics without individual compound-level optimisation. Extraction optimisation is essential for multichemical-origin phenolic plant matrices because different phenolic subclasses (e.g., phenolic acids, flavonoids, tannins) vary widely in polarity, solubility, and stability, requiring particular conditions to maximise their efficient recovery. Inappropriate extraction parameters may lead to the selective loss, under-evaluation, or degradation of key bioactive compounds, thereby reducing both the yield and biological activity of the extract [31,32]. Furthermore, precise optimisation ensures a more complete and reproducible phytochemical profile, which is critical for both scientific and industrial applications.

The results show that the extraction of phenolic compounds from plant material is highly dependent on several parameters, particularly solvent concentration, solvent ratio, and extraction time [33]. Properly selecting these factors is crucial to ensure a high content of phenolic compounds and to obtain extracts with strong antioxidant activity. The results of this study reveal that acetone concentration had the most significant influence on extraction efficiency, significantly affecting the amount of extracted phenolics from different chemical groups. Flavonoids were analysed in two separate categories: flavan-3-ols and related oligomers (condensed tannins), grouped with agrimoniin as high-molecular-weight phenolics; and flavonols and flavones, analysed as low-molecular-weight flavonoid glycosides and aglycones. Secondary metabolites, such as flavonoids, phenolic acids, and tannins, are highly soluble in water, acetone, ethanol, and diethyl ether; however, significant differences in extracted contents can be observed when comparing these solvents [34]. The distinct optimal extraction conditions for phenolic acids (0–5% acetone) versus tannins and flavonoids (34–46% acetone) reflect their differing polarities. While phenolic acids are highly hydrophilic and are extracted efficiently in aqueous solutions, more complex polyphenols, such as agrimoniin, require moderate solvent polarity for optimal solubilisation. Muruzović et al. (2016) determined that the *A. eupatoria* acetone extract showed maximum concentrations of total flavonoids (97.06 mgRU/g), phenols (220.31 mgGA/g), and tannins (207.27 mgGA/g) [14]. The concentrations of these compounds in ethanol and water extracts were half as much, and the diethyl ether extract had the lowest concentration of tannins and phenolics and the greatest antimicrobial and antibiofilm activity [14]. Acetone has also been proven to be the most efficient solvent in extracting polyphenols, particularly condensed tannins, from lychee flowers [35]. Furthermore, certain mixtures of solvents can yield beneficial results for extracting plant matter. The total phenolic compound for black mullberry and blackberry extract was found to be the highest when using acetone/water (70/30 *v*/*v*); for strawberry extracts, the highest phenolic content was found when using acetone/water at different quantities (70/30 and 50/50 *v*/*v*), thus proving that the particular acetone/water ratio is of great importance in determining the extracted contents [36,37]. Ballestros et al. (2015) determined that the best conditions for phenolic extraction from coffee silverskins were achieved when using 60% ethanol in a ratio of 35 mL/g for 30 min at 60–65 °C [38]. This is in agreement with our study, highlighting the critical role of the solvent mixture ratio in determining the efficiency of bioactive compound extraction. Among these, acetone concentration proved to be the most influential factor across all measured groups of phenolic compounds and antioxidant activities. Notably, a 50% acetone concentration consistently led to the highest yields of total phenolics (up to 121.95 mg/g), tannins (17.76 mg/g), flavonoids (10.83 mg/g), and agrimoniin (7.37 mg/g), along with peak antioxidant activities, including radical scavenging (1553.21 µmol/g) and reducing capacities (653.72 µmol/g).

The dominance of agrimoniin in the optimised extracts (27% of total phenolics) highlights its potential as a pharmacologically active marker for quality control. Agrimoniin is a high-molecular-weight ellagitannin found primarily in *Agrimonia* L. species and other plants of the *Rosoideae* subfamily [23,39]. Agrimoniin is considered a chemotaxonomic marker in the species of *Agrimonia* L., *Fragaria* L., and *Potentilla* L. and accounts for 37.5, 11.1, and 32.8 mg/g of dry weight, respectively [39]. Under optimised conditions, we achieved an agrimoniin yield of 9159.46 ± 741.35 µg/g, which is among the higher reported values for *A. eupatoria* extracts. This value significantly exceeds published yields ranging from 2070 to 5430 µg/g [40], demonstrating the selected parameters’ effectiveness in targeting high-molecular-weight ellagitannins. Indeed, the amount of agrimoniin varies significantly from 6.3 up to 207 mg/g depending on solvent selection, with the greatest values obtained when using acetone [23]. Various studies have determined the potent antioxidant, pro-apoptotic, anticancer, antidiabetic, and anti-inflammatory properties of agrimoniin [39,41,42,43,44]. While detailed pharmacokinetic data in humans are limited, preclinical studies suggest that agrimoniin, like other ellagitannins, undergoes hydrolysis in the gut to release ellagic acid, which is further metabolised by the gut microbiota into bioavailable urolithins, expressing systemic anti-inflammatory and antioxidant properties [39]. *A. eupatoria* preparations are defined in the EMA’s HMPC monograph with traditional indications for mild diarrhoea, oral diseases, and skin inflammation [45]. While agrimoniin, as a predominant constituent of pharmacopeial-quality agrimony herbs, contributes to tannin content (≥2% expressed as pyrogallol equivalent) [25], correlation analysis further identified agrimoniin as the compound most strongly associated with antioxidant activity, with r values of 0.886 (ABTS) and 0.921 (FRAP). This highlights its role as a major contributor of phenolics and antioxidants. This compound could serve as a therapeutic target in future extraction development.

Flavonoids are the second dominant group of compounds in *Agrimonia* L. species after tannins [40]. Catechin and quercitrin were identified as the dominant compounds in the flavonoid profile, accounting for up to 18% and 9% of the total identified compounds. Catechin, a well-studied flavanol, exhibits potent antioxidant, anti-inflammatory, neuroprotective, and cardioprotective effects by modulating key cellular pathways such as NF-κB and Nrf2, and by scavenging reactive oxygen species [45]. Its ability to influence oxidative stress-related signalling and mitochondrial health contributes to its potential in preventing cardiovascular and neurodegenerative disorders. Similarly, quercitrin has demonstrated a wide range of pharmacological activities, including antioxidant, anti-inflammatory, and wound healing effects, and has shown promise in managing gastrointestinal, cardiovascular, and neurodegenerative diseases [46]. Its glycoside form enhances solubility and bioavailability, making it a valuable component in bioactive plant extracts [47]. The patterns observed among the different compound groups also suggest a strong relationship between the presence of phenolics, particularly tannins and flavonoids, and antioxidant potential. These compounds are major contributors to the antioxidant properties of various plant extracts [48,49,50,51,52,53,54]. This high yield of principal phenolic compounds combined with strong antioxidant capacity (1553.21 µmol TE/g in DPPH and 653.72 µmol TE/g in FRAP assays) confirms the efficiency and relevance of the RSM-based optimisation strategy. Agrimoniin and flavonoid-rich extracts have potential for the treatment of cancers, neurodegenerative diseases, cardiovascular diseases, and inflammatory diseases, as oxidative stress plays a significant role in the mechanisms of these diseases [41,55].

The antioxidant activity of the *A. eupatoria* extracts was evaluated using the ABTS and FRAP assays, both of which are widely used due to their reproducibility [56]. The ABTS assay measures the ability of antioxidants to scavenge the ABTS^•+^ radical cation, exhibiting both hydrophilic and lipophilic radical scavenging capacities through a single-electron transfer mechanism. In contrast, the FRAP assay evaluates the reducing power of antioxidants by measuring their ability to reduce ferric (Fe^3+^) to ferrous (Fe^2+^) ions in an acidic medium, also through single-electron transfer [57]. These antioxidant properties are reflected in physiologically relevant mechanisms. The free radical scavenging ability of phenolic compounds is important in donating electrons and neutralising ROS in biological systems, while the reducing activity indicates the potential to convert reactive metal ions to less harmful states and to support the regeneration of endogenous antioxidant systems [58].

While this study successfully optimised the extraction of phenolic compounds from *Agrimonia eupatoria* and their antioxidant activity using response surface methodology, several aspects could be targets for future research. First, future studies could incorporate the evaluation of total extraction yield to assess the overall process efficiency, especially for industrial applications. Furthermore, the current model included acetone concentration, solvent ratio, and extraction time under ambient temperature conditions; therefore, the temperature parameter could be included to optimise heat-assisted extraction methods [59]. On the other hand, acetone, being an efficient solvent for extraction, can be removed entirely from the final purified extract [60,61]. Future optimisation should therefore target green extraction alternatives. Together, these future directions will enhance the scientific, practical, and therapeutic potential of *Agrimonia eupatoria* studies.

## 5. Conclusions

This study demonstrated that the extraction efficiency of phenolic compounds and the antioxidant activity of *Agrimonia eupatoria* extracts are significantly influenced by acetone concentration, solvent ratio, and extraction time, with acetone concentration having the most prominent effect. Using response surface methodology, we identified optimal extraction conditions: 34–46% acetone, a 95–100:1 solvent ratio, and a 32–45 min extraction time. These conditions yielded high levels of total phenolics and potent antioxidant activity. Under these conditions, the total phenolic content reached 119.52–121.95 mg/g DW, and the antioxidant activity ranged from 1522.09 to 1553.21 µmol TE/g (radical scavenging activity) and from 636.31 to 653.72 µmol TE/g (reducing activity). The extraction efficiency varied among compound groups: phenolic acids showed optimal extraction at 0–5% acetone, with up to 30% loss under general optimal conditions; tannins and flavan-3-ols, including agrimoniin, were best extracted at 36–57% acetone; flavonoids were efficiently extracted under general conditions. These results highlight the notion of compound-specific efficiency, which is especially relevant for the standardisation of extracts in the development of phytopharmaceuticals and nutraceuticals. The findings also confirm that acetone–water mixtures of intermediate polarity are particularly effective in extracting complex phenolic profiles from tannin-rich plants, such as *Agrimonia eupatoria*. However, this study has several limitations. First, the experimental design did not include replicates at the centre point, which precluded conducting a lack-of-fit test to assess model adequacy. Second, the total extraction yield was not measured, which limits insight into the overall process efficiency, especially for scaling purposes. Third, acetone–water mixtures were not compared to water or ethanol, which restricts broader applicability. These aspects should be addressed in future research to further optimise extraction protocols and enhance industrial relevance.

## Figures and Tables

**Table 1:** The experimental extraction conditions.

Extraction No.	Factor A	Factor B	Factor C
	Acetone Concentration (%)	Solvent Ratio	Extraction Time (min)
E-01	50	55	25
E-02	100	100	5
E-03	0	100	5
E-04	50	55	45
E-05	50	100	25
E-06	50	10	25
E-07	0	100	45
E-08	50	55	5
E-09	0	10	45
E-10	100	100	45
E-11	100	55	25
E-12	0	10	5
E-13	100	10	5
E-14	0	55	25
E-15	100	10	45

**Table 2:** ANOVA data of the fit of the model.

Response	Model	*p*-Value
Total phenolic content	quadratic	0.0503
Cumulative amount of phenolic acids	linear	0.0382
Cumulative amount of tannins	quadratic	0.1560
Cumulative amount of flavonoids	quadratic	0.1725
Amount of agrimoniin	quadratic	0.1824
Radical scavenging activity	quadratic	0.0789
Reducing activity	quadratic	0.0060

**Table 3:** The significant data of the model terms.

	Response	
Factors	Cumulative Amount of Phenolic Acids	Reducing Activity
A (acetone concentration)	*p* = 0.0126	*p* = 0.0023
B (solvent ratio)	*p* = 0.2082	*p* = 0.2283
C (extraction time)	*p* = 0.2821	*p* = 0.1084
AB	–	*p* = 0.9401
AC	–	*p* = 0.2089
BC	–	*p* = 0.8997
A^2^	–	*p* = 0.0008
B^2^	–	*p* = 0.6955
C^2^	–	*p* = 0.3675

**Table 4:** ANOVA data of the reduced model.

Response	Model *p*-Value	Factor A *p*-Value	Factor A^2^ *p*-Value
Total phenolic content	<0.0001	0.0009	<0.0001
Cumulative amount of phenolic acids	0.0133	0.0133	–
Cumulative amount of tannins	0.0045	0.1850	0.0020
Cumulative amount of flavonoids	0.0056	0.2128	0.0024
Amount of agrimoniin	0.0017	0.2232	0.0006
Radical scavenging activity	<0.0001	0.0014	<0.0001
Reducing activity	<0.0001	0.0002	<0.0001

**Table 5:** The statistical data and mathematical equations of significant models.

Response	Adjusted R^2^	Predicted R^2^	Δ R^2^	Adeq Precision	The Equations (Actual Factors ^1^)
Total phenolic content	0.8100	0.7456	0.06	11.1	Y = 66.75 + 2.026X − 0.025X^2^
Cumulative amount of phenolic acids	0.3395	0.1948	0.14	4.9	Y = 2.798 − 0.018X
Cumulative amount of tannins	0.5257	0.3647	0.16	5.8	Y = 6.74 + 0.276X − 0.003X^2^
Cumulative amount of flavonoids	0.5078	0.3408	0.17	5.6	Y = 4.24 + 0.173X − 0.002X^2^
Amount of agrimoniin	0.5982	0.4619	0.14	6.5	Y = 2.03 + 0.145X − 0.002X^2^
Radical scavenging activity	0.7881	0.7162	0.07	10.4	Y = 871.16 + 28.31X − 0.35X^2^
Reducing activity	0.8650	0.8191	0.05	13.5	Y = 345.05 + 11.72X − 0.15X^2^

^1^ X and X^2^ are acetone concentration. Y is the appropriate response.

## Data Availability

The original contributions presented in this study are included in the article. Further inquiries can be directed to the corresponding author(s).

## Supplementary Materials

The following supporting information can be downloaded at: https://www.mdpi.com/article/10.3390/antiox14070831/s1, Table S1: HPLC-PDA method validation parameters.
