# Supplementary material for: Optimisation of Phenolic Compound Extraction from Agrimonia eupatoria L. Using Response Surface Methodology for Enhanced Yield of Different Phenolics and Maximised Antioxidant Activity
Source: Antioxidants (Basel). 2025 Jul 7;14(7):831. doi: 10.3390/antiox14070831 (PMC12291865; doi:10.3390/antiox14070831)
Supplement: Supplementary file 1 [file antioxidants-14-00831-s001.zip › antioxidants-3695661-supplementary.pdf]

Table. S1. HPLC-PDA method validation parameters.

Compound	calibration curve	coefficient of determination (R^2)	linearity range, (µg/ml)	LOD, (µg/ml)	LOQ, (µg/ml)	Recovery%	Repeatability %	Intermediate precision %
Neochlorogenic acid	y=25600x+17300	0.99944	5-80	0.74	1.29	100.5	0.45	1.21
Chlorogenic acid	y=47700x+2650	0.99992	2.5-50	0.64	1.95	99.1	1.17	1.32
4-O-caffeoylquinic acid	y=60200x-16200	0.99996	2.5-50	0.46	1.38	100.7	1.36	2.52
Ellagic acid	y=201000x-113000	0.99951	6.25-200	0.69	2.09	100.4	1.11	2.08
Catechin	y=9460x+7290	0.99938	2.5-40	0.34	1.08	107.2	0.77	1.6
Epicatechin	y=11000x+1660	0.99994	2.5-40	0.43	1.3	97.6	1.22	2.06
EGCG	y=31800x+6380	0.99999	3.125-100	0.48	1.44	98.2	1.47	2.32
GCG	y=21100x+84400	0.99918	3.125-50	1.22	2.9	102.5	1.82	1.33
Procyanidin B1	y=10700x-5100	0.99965	6.25-100	0.69	1.15	91.5	1.17	2.63
Procyanidin B2	y=9680x-8300	0.99991	6.25-200	0.68	1.17	107.2	0.89	1.94
Agrimoniin	y=25700x-47500	0.99922	1.25-100	0.54	1.38	95.4	1.6	2.23
p-coumaric acid	y=140000x-19100	0.99998	3.125-50	0.31	0.95	98.2	1.44	1.3
Hyperoside	y=43700x+3370	0.99996	2.5-40	0.32	0.98	100.9	0.96	2.25
Luteolin-7-glucoside	y=54300x+1040	0.99998	2.5-40	0.26	0.79	100.5	1.15	1.1
Luteolin-7-glucuronide	y=49300x+20600	0.99943	1.5-50	0.86	1.64	94.2	1.93	2.18
Isovitexin	y=40300x+27400	0.99957	4.5-50	0.62	0.92	98.7	1.24	1.83
Quercetin-3-malonyl-glucoside	y=23700x-46000	0.99999	3.1-200	0.98	1.01	100.7	1.08	2.01
Isoquercitrin	y=38400x+74800	0.99983	12.5-200	0.73	1.31	105.5	0.52	2.27
Astragalin	y=41600x+435	0.99999	3.12-50	0.69	0.77	107.5	1.15	2.64
Quercitrin	y=30500x+1380	0.99999	3.12-50	0.23	0.70	98.7	1.41	1.12
Tiliroside	y=44200x-1180	0.99999	2.5-40	0.09	0.30	97.6	0.65	1.28
Quercetin	y=61900x-10000	0.99999	3.125-50	0.27	0.83	99.4	1.27	1.48
